# PSMA-Based Radiopharmaceuticals in Prostate Cancer Theranostics: Imaging, Clinical Advances, and Future Directions

**DOI:** 10.3390/cancers18020234

**Published:** 2026-01-12

**Authors:** Ali Cahid Civelek

**Affiliations:** Division of Nuclear Medicine and Molecular Imaging and Theranostics, Department of Radiology and Radiation Sciences, The Johns Hopkins University School of Medicine, Baltimore, MD 21287, USA; acivele1@jhmi.edu

**Keywords:** prostate cancer, PSA, PSMA, mCRPC, [^68^Ga]Ga, ^18^F-PyL-PSMA PET/CT, Pylarify (Piflufolastat-F18), targeted radioligand therapy, theranostics, [^177^Lu]Lu-PSMA-617, Pluvicto, radioligand therapy (RLT), AI, radiomics

## Abstract

Prostate cancer remains one of the most common malignancies in men worldwide, with incidence and mortality steadily increasing across diverse populations. While early detection and radical prostatectomy can achieve durable control in a subset of patients, approximately 40% of men will ultimately experience biochemical recurrence often in the absence of clinically detectable disease. Conventional imaging approaches—CT, MRI, and bone scintigraphy—have limited sensitivity for early relapses, frequently leading to delayed diagnosis and suboptimal treatment planning. The discovery of prostate-specific membrane antigen (PSMA) in 1987 and its subsequent clinical translation into positron emission tomography (PET) imaging with [^68^Ga]Ga-PSMA-11 in 2012, followed by U.S. FDA approval in 2020, has transformed the landscape of prostate cancer imaging. PSMA PET has demonstrated superior accuracy over conventional imaging, as highlighted in the landmark proPSMA trial and now serves as the foundation for theranostic approaches that integrate diagnostic imaging with targeted radioligand therapy. The clinical approval of [^177^Lu]Lu-PSMA-617 (Pluvicto^®^: (lutetium Lu 177 vipivotide tetraxetan, Advanced Accelerator Applications USA, Inc., a Novartis company) has established targeted radioligand therapy as a viable option for men with metastatic castration-resistant prostate cancer, extending survival in patients with limited alternatives. Emerging strategies, including next-generation ligands with improved tumor uptake and altered clearance pathways, as well as the integration of artificial intelligence for imaging quantification, are poised to further refine patient selection, dosimetry, and treatment outcomes. This review highlights the evolution of PSMA-based imaging and therapy, discusses current clinical applications and limitations, and outlines future directions for optimizing theranostic strategies in prostate cancer care.

## Simple Summary

Significant advancements have been made since the approval of the Prostate-Specific Antigen (PSA) test in 1986 and the identification of Prostate-Specific Membrane Antigen (PSMA) in 1987, leading to the introduction of the first-in-human [^68^Ga]Ga-PSMA-11 PET imaging. Despite these developments, prostate cancer continues to be a leading cause of cancer-related mortality among men worldwide. This ongoing challenge largely stems from the limitations of current PSMA imaging techniques, which can miss a subset of malignant lesions, resulting in variable patient outcomes. However, recent innovations in theranostic and chemotherapeutic agents, along with the implementation of immunotherapy, are improving patient outcomes and survival rates. Additionally, advancements in imaging technologies—including hardware improvements, the integration of artificial intelligence (AI), and radiomics—are significantly enhancing the diagnostic and therapeutic landscape. This review article aims to summarize recent progress in both diagnostic and therapeutic domains while discussing relevant ongoing clinical trials. It is important to note that this summary may not cover all recent developments, as the rapid pace of advancements in multimodal approaches within this field may have led to some progress being overlooked.

## 1. Introduction

Prostate cancer (PCa) is the second most frequently diagnosed cancer in men and a leading cause of cancer-related mortality worldwide. According to recent estimates from the U.S. National Cancer Center, the incidence and death rates of prostate cancer continue to rise annually, reflecting both an aging population and increased detection. Although many prostate cancers follow an indolent course, others exhibit aggressive biology that necessitates intensive treatment, including radical prostatectomy, androgen deprivation therapy, and systemic chemotherapy [1].

A major challenge in the clinical management of prostate cancer is the occurrence of biochemical recurrence (BCR) following definitive therapy. Approximately 40% of patients who undergo radical prostatectomy eventually develop BCR [1], typically defined as a sustained rise in prostate-specific antigen (PSA) to ≥0.2 ng/mL confirmed by subsequent testing. Notably, only 10–20% of these patients will have clinically detectable recurrence on conventional imaging [2]. This discrepancy underscores the limitations of standard radiographic methods [3] in early disease detection and highlights the urgent need for more sensitive and specific tools.

The evolution of prostate cancer diagnostics has been shaped by key milestones. The introduction of bone scintigraphy in the early 1970s allowed detection of skeletal metastases [3], while the approval of the PSA test in 1986 revolutionized monitoring for recurrence and treatment response. The discovery of prostate-specific membrane antigen (PSMA) in 1987 represented another pivotal advance, though it would take decades before its clinical potential was fully realized. The translation of PSMA into PET imaging, particularly with the approval of [^68^Ga]Ga-PSMA-11, has provided a transformative leap forward in both staging and recurrence detection, with average detection efficiencies reported near 80% [4,5].

Despite this progress, current PSMA imaging still misses a subset of malignant lesions, and there remains considerable heterogeneity in patient outcomes. Moreover, the rapid adoption of PSMA-targeted therapies has introduced new challenges related to patient selection, dosing strategies, and management of resistance. Against this backdrop, the concept of Theranostics, where diagnostic imaging guides the application of targeted radioligand therapy—has emerged as a cornerstone of precision oncology in prostate cancer.

Theranostic Concept ^177^Lu-‘Pluvicto’ as the radioligand therapy for prostate cancer Theranostics Agent: The term “theranostic” combines therapy and diagnostics into a single concept. In a theranostic approach, a patient is first imaged using a targeted PET (positron emission tomography) or SPECT (single-photon emission computed tomography) tracer, which reveals the distribution of specific tumor receptors. If the tumor demonstrates sufficient uptake of the biomarker, the patient may be selected for radionuclide therapy using a corresponding radiotherapeutic tracer with a similar structure.

This process begins by evaluating the binding of the tracer and the expression of PSMA in the tumor to ensure the patient is suitable for treatment. PSMA-617 can be labeled with ^68^Ga (or ^18^F) for imaging or with ^177^Lu for therapy, providing a cohesive strategy for both diagnosis and treatment. In targeted Radionuclide therapy (RLT), ^177^Lu delivers radiation specifically to tumor cells while minimizing damage to healthy tissue. After binding to the PSMA receptor, [^177^Lu]Lu-PSMA-617 is internalized into PSMA-positive cells, leading to cell death due to DNA damage caused by emitted high-energy electrons.

While β-emitters are commonly used in RLT because of their low linear energy transfer (LET), alpha emitters are emerging as promising alternatives for smaller tumors due to their high LET and shorter range. However, further studies are necessary. The effectiveness of [^177^Lu]Lu-PSMA-617 for prostate cancer treatment has been validated in multiple clinical trials, leading to FDA approval [6].

### 1.1. Advances in Imaging

The evolution of prostate cancer imaging reflects the broader trajectory of oncologic imaging: from indirect, low-sensitivity methods to highly specific molecular approaches that can both detect and characterize disease. Historically, bone scintigraphy using ^99m^Tc-labeled phosphates and diphosphonates, first proposed in 1971, represented the standard of care for detecting skeletal metastases [3]. While valuable, these methods lacked sensitivity in identifying early or small-volume metastatic deposits, and they provided no insight into soft-tissue disease. Computed tomography (CT) and magnetic resonance imaging (MRI) expanded diagnostic capabilities but similarly demonstrated limited accuracy in staging and in localizing biochemical recurrence at low PSA levels. Table 1 provides a Comparative Overview of Imaging Modalities in Prostate Cancer.

The introduction of prostate-specific antigen (PSA) testing in the 1980s significantly enhanced disease monitoring, enabling earlier detection of recurrence. However, PSA alone cannot localize disease and often leads to therapeutic uncertainty when conventional imaging remains negative. This gap set the stage for the integration of prostate-specific membrane antigen (PSMA) into clinical practice.

**Figure 1:**
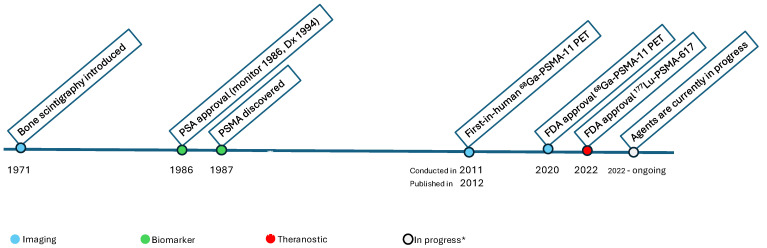
Timeline of Milestones in Prostate Cancer Imaging and Theranostics. * Agents are currently in progress including subtypes of PSMA imaging agents, Theranostics, including alpha (α) emitting agents. The first-in-human [^68^Ga]Ga-PSMA-11 PET was conducted in 2011, followed by numerous publications from 2012 onward [7].

PSMA is a type II transmembrane glycoprotein with high expression in prostate cancer cells, particularly in advanced and castration-resistant disease. Its discovery in 1987, and subsequent exploitation as a molecular target, culminated in the clinical application of [^68^Ga]Ga-PSMA-11 PET/CT in 2012, which was later approved by the U.S. FDA in 2020 [5]. Figure 1. This development marked a turning point in prostate cancer imaging, enabling unprecedented sensitivity and specificity for both primary staging and detection of recurrence. Average diagnostic performance across multiple series has been estimated at approximately 80%, representing a substantial improvement over traditional modality.

**Figure 2:**
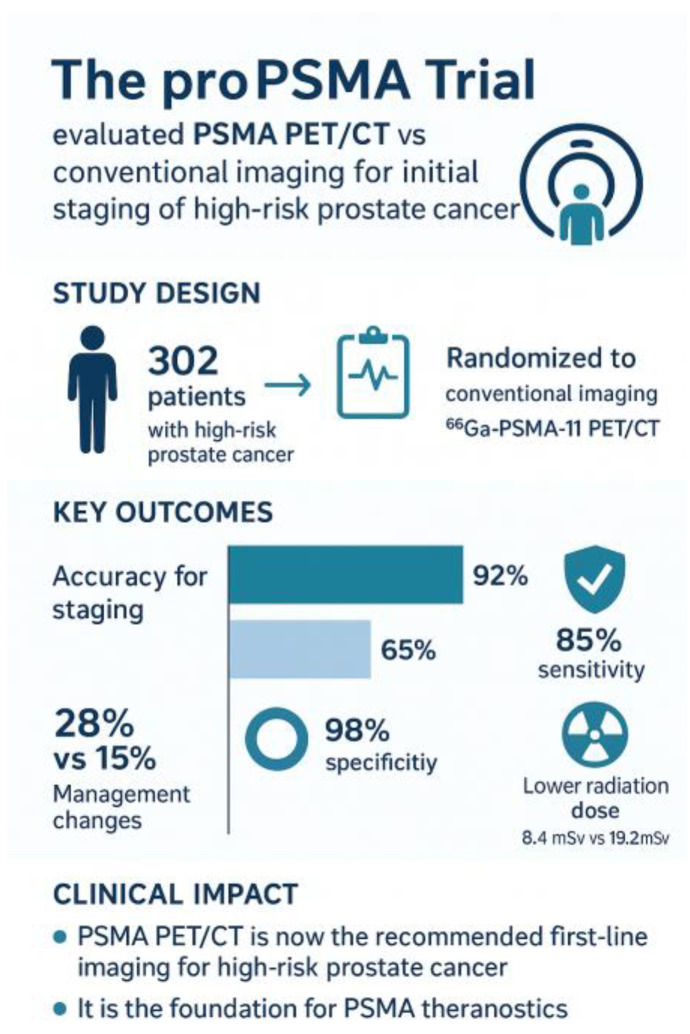
proPSMA-Graphical summary.

The proPSMA trial, a prospective randomized multicenter study published in The Lancet in 2020, provided compelling evidence for the superiority of PSMA PET/CT over conventional imaging [8]. In this study of 302 men with high-risk, biopsy-proven prostate cancer, participants randomized to PSMA PET/CT achieved markedly higher accuracy in detecting nodal and distant metastases compared with those undergoing CT and bone scintigraphy. Importantly, PSMA PET altered clinical management in a significant proportion of patients, underscoring its value as a replacement for standard imaging in staging high-risk disease [8]—Figure 2.

Case-based evidence further demonstrates the clinical impact of PSMA PET. For example, patients with negative conventional imaging but positive PSMA PET findings have been shown to harbor metastatic deposits detectable only through PSMA-targeted methods. Longitudinal follow-up of such patients often confirms true-positive findings, with regression or sclerosis of lesions after systemic treatment and corresponding declines in PSA. These observations illustrate how PSMA PET can unmask otherwise occult disease, thereby guiding more appropriate therapy [6].

The development of additional tracers has expanded the armamentarium of PSMA-targeted imaging. Agents such as ^18^F-DCFPyL (Pylarify) and ^18^F-PSMA-1007, approved in 2021, provide advantages in production scalability and imaging characteristics, including reduced urinary excretion compared with gallium-based tracers. Novel ligands, such as ^18^F-rhPSMA-7.3, are under investigation for their potential to improve detection rates, particularly in post-treatment settings where tracer clearance pathways significantly affect interpretability (Table 2).

#### 1.1.1. The Transformative Impact of Advancements in Hardware Technology

Advancements in technology are significantly enhancing the performance and efficiency of medical imaging, fundamentally transforming how physicians engage with and utilize this technology in their daily practice to improve patient outcomes. Emerging imaging technologies further enhance diagnostic yield.

Large axial field-of-view (FOV) PET/CT systems equipped with digital silicon photomultipliers enable whole-body coverage with higher sensitivity and lower radiation exposure [14].

The standard axial field of view (SAFOV) for PET/CT scanners typically ranges from 15 to 28 cm, and these scanners usually employ analog photomultiplier tubes (PMTs). In contrast, the newer Long Axial Field of View (LAFOV) PET/CT scanners feature an axial field of view ranging from 10.6 cm (as seen in the Biograph Vision Quadra) to 194 cm (as found in the uExplorer), utilizing Silicon Photomultipliers (SiPMs). Such advancements in PET/CT scanner technology greatly improve diagnostic accuracy, likely leading to better patient outcomes.

The primary challenge in current imaging practices using standard PET/CT scanners is the detection of small PSMA-positive lesions that exhibit low tumor activity, as it is the case in previously treated biochemical recurrence metastatic prostate cancer (BCR mPc) patients with PSA < 0.2 ng/mL. Often, the signals from these small recurrent or metastatic lesions are obscured by significant statistical noise. However, new Long Axial Field of View (LAFOV) PET/CT scanners that utilize silicon-based detectors offer significantly improved performance compared to standard axial field of view scanners. These advanced systems have enhanced sensitivity and provide a superior signal-to-noise ratio (SNR) and contrast-to-noise ratio (CNR), allowing for effective suppression of background noise. This improvement enables the differentiation of subtle signals that would otherwise be indistinguishable in standard imaging conditions.

For instance, Wang et al. reported that when a large axial field of view (LAFOV) PET/CT scanner was used for imaging patients with prostate-specific antigen (PSA) levels greater than 0.2 ng/mL, the detection rate for biochemical recurrence (BCR) of prostate cancer lesions using ^68^Ga-PSMA-11 increased to 91%. In contrast, the detection rate was only 74% with a standard axial field of view PET/CT scanner (*p* = 0.003) [14].

Additionally, in patients with PSA levels below 0.2 ng/mL, the BCR of prostate cancer lesion detection using [^68^Ga]Ga-PSMA-11 with LAFOV PET/CT rose to 73.8%. This was significantly higher than the 43.8% detection rate achieved with a standard field of view PET/CT scanner [15].

Early and accurate localization and detection of recurrent or metastatic prostate cancer lesions are crucial for determining therapy and securing a favorable outcome for the patient [16] (Table S1).

Also, in a HEAD-to-head comparative analysis, the Biograph Vision Quadra demonstrated a significantly reduced acquisition time of under 2 min, while still achieving diagnostic image quality comparable to that of a conventional standard axial FOV scanner, which typically requires 16 min for a mid-skull to mid-thigh PET/CT image acquisition [17].

#### 1.1.2. PET/MR

The initial clinical systems for PET/MRI were introduced in 2010, with the first PSMA PET/MRI scans likely occurring around 2013. The earliest comparative research examining PSMA PET/MRI versus PET/CT was published in 2016. The effectiveness of Theranostics is contingent not only on the specificity of the ligands but also on the advanced imaging technologies employed to optimize their application, particularly the combination of PET and MRI modalities in PET/MRI. The utilization of PSMA PET/MRI, in conjunction with PET/CT and relevant quantitative parameters, is crucial for the management of castration-resistant prostate cancer and other urogenital malignancies [18,19]. Emerging preliminary studies suggest that hybrid PET/MRI systems, when paired with radiolabeled PSMA such as [^68^Ga]Ga-PSMA-11, may provide at least comparable enhancements in diagnostic accuracy for prostate cancer detection. As these technologies continue to advance, PSMA-targeted imaging with hybrid PET/MRI is expected to integrate into standard diagnostic evaluation and management frameworks for patients with prostate cancer [20].

#### 1.1.3. Advancements in Image Reconstruction Algorithms

**Figure 3:**
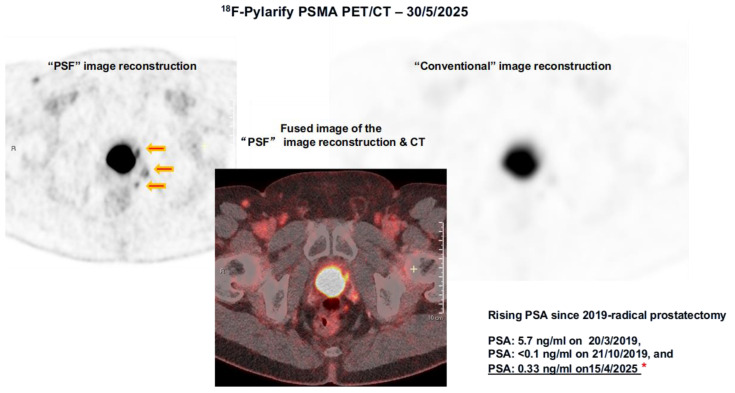
PSF vs. Conventional Image Reconstruction of the same ^18^F-Pylarify PSMA PET/CT. Three small PSMA-avid lesions adjacent to the bladder identified on the PSF-reconstructed PET/CT images (red arrows) are not discernible on the conventional reconstruction of the same dataset. Without this advanced imaging technique, these lesions would have likely gone undetected. The red asterisk indicates the patient’s PSA level, recorded in close temporal relation to the PSMA PET/CT scan. Together, these advances underscore the transformative role of PSMA-based imaging in prostate cancer management. By providing both higher diagnostic accuracy and biologically relevant insights, PSMA PET has not only improved patient stratification but also established the foundation for theranostic applications, where diagnostic imaging directly informs targeted radioligand therapy.

Image reconstruction algorithms, such as point spread function (PSF) modeling, improve lesion contrast and quantitative accuracy compared with conventional methods [21]. Figure 3 shows a patient with an increasing PSA level after undergoing prostatectomy. PSF reconstruction identified highly suspicious areas of uptake in the prostate bed and regional lymph nodes that were either less noticeable or not visible on the PET/CT images processed with a standard reconstruction algorithm. This finding significantly impacted decisions regarding salvage treatment.

### 1.2. Theranostic Concepts

The integration of PSMA-targeted imaging and therapy—known as Theranostics—represents a major advance in precision oncology for prostate cancer [6,20]. This approach links diagnostic molecular imaging directly to targeted radioligand therapy, ensuring that the same biomarker used to detect disease is also exploited for treatment.

The most widely adopted example is [^177^Lu]Lu-PSMA-617 (Pluvicto), which received FDA approval in 2022 for men with PSMA-positive metastatic castration-resistant prostate cancer (mCRPC) [19,22]. Patients considered for therapy must first undergo PSMA PET imaging to confirm adequate tumor uptake, with lesions demonstrating greater tracer accumulation than normal liver serving as a threshold for eligibility. Standard therapy consists of intravenous administration of 7.4 GBq every six weeks for up to six cycles [23,24] (Table S1).

**Figure 4:**
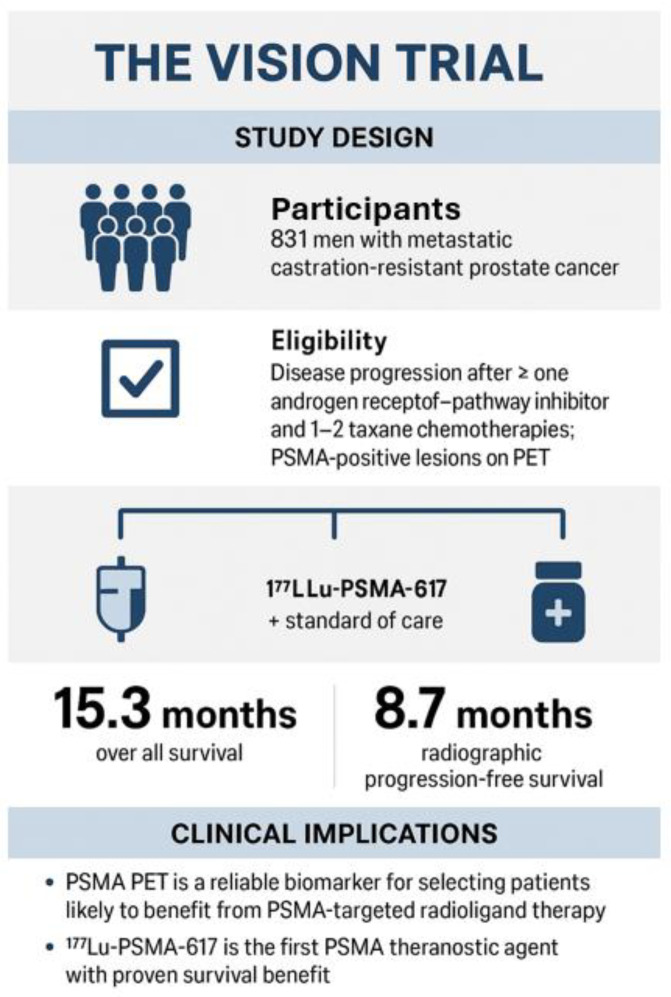
The VISION trial, Sartor et al., NEJM 2021 [23], a pivotal, international, phase 3 randomized controlled trial that established the clinical benefit of ^177^Lu-PSMA-617 radioligand therapy in advanced prostate cancer, with a cohort of 831 men with mCRPC. The rigid eligibility criteria required disease progression after ≥1 androgen receptor–pathway inhibitor (e.g., enzalutamide, abiraterone) and 1–2 taxane chemotherapy regimens, with PSMA-positive lesions confirmed by PET imaging, which validated PSMA theranostics as a standard of care in advanced prostate cancer, extending both survival and quality of life for men with limited therapeutic options. Based on results from the VISION trial, The U.S. FDA first approved Pluvicto on 23 March 2022, for adult patients with PSMA-positive mCRPC who had previously received both ARPI therapy and taxane-based chemotherapy. Since then, the use of [^177^Lu]Lu-PSMA-617 radioligand therapy has become more flexible. The most recent approval for [^177^Lu]Lu-PSMA-617 (Pluvicto) was granted by the FDA on 28 March 2025. This expanded labeling permits Pluvicto to be administered earlier in the disease progression, specifically for patients with metastatic castration-resistant prostate cancer (mCRPC) who have been treated with androgen receptor pathway inhibitors (ARPI) but have not undergone chemotherapy.

The pivotal VISION trial demonstrated that [^177^Lu]Lu-PSMA-617 improves overall survival and radiographic progression-free survival in heavily pretreated patients, establishing targeted radioligand therapy as a new standard of care in advanced prostate cancer [23]. Figure 4. Common adverse effects include fatigue, dry mouth, and hematologic suppression, but these are generally manageable with careful monitoring of renal function and blood counts. Table 3.

Beyond mCRPC, Theranostics is rapidly expanding into earlier disease stages [25,26]. Ongoing studies are exploring neoadjuvant use in high-risk patients, integration with androgen receptor pathway inhibitors, and combination with external beam radiotherapy. The goal is to leverage PSMA-targeted therapy not only for palliation but also as a component of potentially curative strategies [25,26,27,28].

At its core, Theranostics exemplifies personalized medicine. By aligning diagnosis and therapy through a single molecular target, clinicians can select the right patients, deliver treatment more effectively, and monitor response with unprecedented accuracy [23,29].

## 2. Emerging Radioligand Therapies

**Figure 5:**
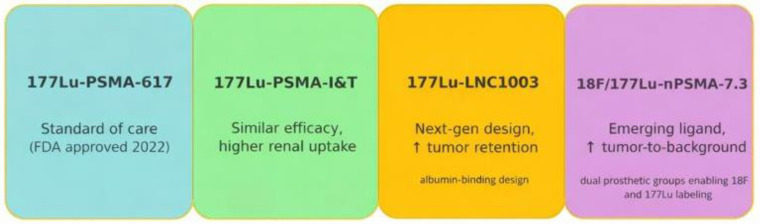
Emerging Radioligands Pipeline. Independent development paths of PSMA ligands. The timeline for PSMA I&T is about 2015 [7]. The arrows (↑) in the figure indicate increased tumor retention and an increased tumor-to-background ratio.

While [^177^Lu]Lu-PSMA-617 has set the benchmark for prostate cancer Theranostics, newer radioligand therapies are under development to address its limitations. One challenge with PSMA-617 is its relatively rapid clearance from the bloodstream, which can limit tumor uptake and necessitate repeated dosing. Figure 5 provides a summary of the pipeline for emerging PSMA theranostic radioligands.

Alternative agents, such as [^177^Lu]Lu-PSMA-I&T (also known as zadavotide guraxetan [(^177^Lu) zadavotide guraxetan is under clinical development by Eli Lilly and Co and currently in Phase III for Metastatic Castration-Resistant Prostate Cancer (mCRPC)), have been widely used in Europe and demonstrate similar therapeutic efficacy. However, PSMA-I&T has higher kidney uptake compared with PSMA-617, raising potential concerns about renal toxicity [24,30,31,32]. Efforts are ongoing to optimize ligands with more favorable biodistribution profiles.

Next-generation compounds, such as [^177^Lu]Lu-LNC1003, are designed with prolonged circulation times, achieving substantially higher absorbed tumor doses in preclinical studies compared to PSMA-617. Early results suggest these may improve treatment efficacy while reducing the need for multiple administrations [22].

Fluorine-labeled ligands like ^18^F/[^177^Lu]Lu-rhPSMA-7.3 represent another promising direction. In comparative studies, rhPSMA-7.3 demonstrated greater tumor retention and a trend toward longer survival compared with earlier ligands [30]. Importantly, this agent also shows reduced renal excretion, which could translate into improved detection in the pelvis and potentially lower renal toxicity during therapy [7,30], Figure 5.

Future trends in PSMA theranostics are increasingly focusing on hormone-sensitive patients and integrating these therapies into the neoadjuvant treatment approach, and in high-risk patients with non-metastatic hormone sensitive prostate cancer patients (nmHSPC) Table 4 and Table 5. As a result, the target population for PSMA theranostics is likely to encompass individuals with significantly longer expected survival rates. In this context, minimizing radiation exposure and kidney toxicity will become crucial, especially as we progress towards treating younger patients and those diagnosed at earlier stages of metastasis. This shift highlights the importance of developing safer treatment options for a population that will benefit from prolonged care and improved outcomes. Table 3.

The emergence of multiple radioligand options raises the possibility of tailoring treatment to patient-specific factors such as renal function, tumor burden, and prior therapies. Just as oncology has moved toward biomarker-driven systemic therapy, prostate cancer Theranostics is evolving into a field where ligand choice may be individualized for optimal efficacy and safety.

In practice, the growing diversity of available ligands will enable more personalized therapeutic strategies, mirroring the precision medicine approaches already well established in systemic oncology.

## 3. Integration with Imaging Advances and Artificial Intelligence

Recent advances in PET hardware, including large axial field-of-view systems with digital detectors, have improved whole-body imaging by enhancing sensitivity and speeding up acquisition times. This leads to better detection of small or early metastatic deposits, aiding in more accurate staging and treatment planning [14,15,17]. Improvements in image reconstruction techniques, such as advanced point spread function modeling, enhance lesion contrast and quantitative accuracy, which can impact therapeutic decisions, such as eligibility for salvage radiation or systemic therapy. Figure 3.

Artificial intelligence (AI) emerges as a powerful tool to complement these hardware and software advances. Fully automated methods now allow volumetric assessment of tumor burden on PSMA PET, providing objective and reproducible metrics that can predict survival after radioligand therapy [42,43]. By moving beyond qualitative interpretation, AI-driven analysis offers the potential to stratify patients more precisely, identify those most likely to benefit from therapy, and monitor treatment response over time [42,43].

For instance, Dr. Feng Wang in a recent study demonstrated that the Fully Automated Volumetric Assessment of Tumor Burden using Artificial Intelligence with ^68^Ga-PSMA-11 PET predicts survival after [^177^Lu]Lu-PSMA therapy in patients with metastatic castration-resistant prostate cancer. This research addresses an important and timely topic at the intersection of molecular imaging, artificial intelligence, and Theranostics in the treatment of advanced prostate cancer. The introduction of a multi-task segmentation network represents a significant advancement aimed at reducing false positives while incorporating anatomical context, thereby enhancing its clinical applicability. This improvement is achieved through a thorough evaluation of segmentation quality, consistent reliability among different assessors, and high intra-rater agreement [42].

**Figure 6:**
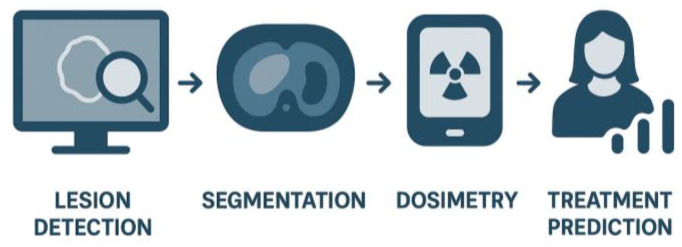
AI Integration in PSMA Theranostics.

Together, these innovations are reshaping the clinical application of PSMA Theranostics. The combination of high-performance imaging platforms, advanced reconstruction, and AI-based semi-quantification provides clinicians with a more reliable foundation for decision-making. Ultimately, this integration will help ensure that radioligand therapies are delivered to the right patients, at the right time, with the greatest likelihood of success (Figure 6).

### 3.1. Radiomics

The term coined by Lambin et al. in 2012 [44] refers to the extraction of large amounts of quantitative data from medical images that may not be recognized or quantified by the human eye, even an expertly trained one, and which is ideally reproducible.

Radiomics is a field of medical imaging that extracts quantitative features from standard medical images, such as CT, MRI, or PET scans, using advanced computational methods (Figure 6).

The core idea is that instead of just looking at images visually (as a radiologist does), radiomics converts them into large sets of numerical data. These numbers describe characteristics like texture, shape, intensity, heterogeneity, and spatial relationships within a tumor or tissue. Why is this useful? Tumors may look similar on a scan, but their underlying biology (e.g., aggressiveness, treatment response) can differ.

Radiomics features can capture subtle patterns that the human eye cannot see.

When combined with machine learning and clinical/genomic data, radiomics can help in: Diagnosis (distinguishing benign vs. malignant lesions), Prognosis (predicting disease course), Therapy response prediction (who will respond to chemo, radiation, immunotherapy, or theranostics), and Precision medicine (personalized treatment planning).

As a simple analogy, think of radiomics as turning a CT/MRI/PET scan into a “digital biopsy”—extracting microscopic-level information from macroscopic images without physically sampling the tissue.

PET imaging is essential for diagnosis, staging, and therapy guidance especially for Theranostics such as PSMA PET/CT and ^177^Lu-Pluvicto treatment.

Radiomics adds a layer by extracting quantitative imaging features beyond standard PET metrics, such as SUVmax.

Current Evidence indicates that research is emerging but remains limited, as most studies are small cohorts or retrospective. However, Early studies suggest that PET radiomics improves the prediction of tumor aggressiveness, provides non-invasive insights into tumor biology, and supports precision medicine approaches in mRPC management.

Radiomics uses computer algorithms to extract and analyze a large number of quantitative features from radiological images, drawing on multiple scientific disciplines, such as Artificial Intelligence (AI), Machine Learning (ML), Deep Learning (DL), and Data analytics and Computer Vision [45].

### 3.2. Future Directions of Radiomics in PCa

Standardization of imaging protocols, features, and analysis methods will improve reliability and clinical adoption.

Integration with artificial intelligence and deep learning will enable automated segmentation, analysis, and more robust predictive models.

Multicenter prospective validation studies and collaborative efforts are necessary to advance patient care in prostate cancer (PCa).

Ultimately, radiomics may enable “virtual biopsy,” improving non-invasive tumor characterization and guiding precision medicine.

Combining radiomics with clinical, biochemical, and genetic data along with transcriptomics, proteomics, and metabolomics promises more precise and personalized diagnosis & treatment [45,46].

AI-Enhanced Radiomics Ecosystem Integrates radiomics with clinical, dosimetric, and genomic data; machine- and deep-learning frameworks for prediction and treatment planning; and Harmonization (ComBat) and IBSI standards to improve reproducibility [47].

In Summary, Radiomics enhances diagnosis, risk stratification, and theragnostic planning, integrates with PSMA PET, mpMRI, and genomics, and drives precision medicine. Reproducibility and validation remain the bridge to clinical adoption [47,48,49,50,51]. (Table 6).

## 4. Clinical Challenges and Future Directions

Despite remarkable progress, several challenges remain in optimizing PSMA Theranostics for prostate cancer [55].

Biochemical recurrence (BCR) continues to be one of the most pressing clinical dilemmas [1,4,5]. Approximately 40% of patients treated with radical prostatectomy will eventually experience BCR, typically defined as a confirmed PSA rise above 0.2 ng/mL. Yet, conventional imaging often fails to localize recurrence at these low PSA levels, leaving clinicians uncertain about when and where to intervene. PSMA PET has significantly improved detection in this context, frequently identifying disease in patients previously classified as non-metastatic. However, how best to treat patients with oligometastatic or polymetastatic findings on PSMA PET remains unsettled. Establishing evidence-based strategies for this growing group is a critical future need.

Another limitation lies in the prevailing “one-size-fits-all” dosing paradigm. Current radioligand therapy protocols administer fixed activities of [^177^Lu]Lu-PSMA-617 regardless of patient-specific variables such as tumor burden, renal function, or percent tracer uptake by the malignant tumor. While practical, this approach risks undertreating some patients while exposing others to unnecessary toxicity. Individualized dosimetry—where doses are tailored to maximize tumor dose while sparing normal organs—offers a more rational solution. Streamlining these calculations into routine practice will be essential for realizing the full potential of Theranostics [32].

**Figure 7:**
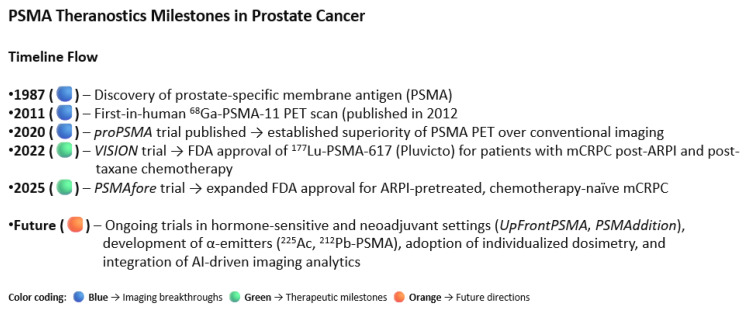
This visual summary illustrates the evolution of prostate-specific membrane antigen (PSMA) imaging and theranostics in prostate cancer.

The future directions of the PSMA Theranostics are summarized in Figure 7. Beginning with the discovery of PSMA in 1987 and the first-in-human [^68^Ga]Ga-PSMA-11 PET scan in 2011, PSMA-targeted diagnostics rapidly advanced, culminating in the *proPSMA* trial (2020), which demonstrated the superiority of PSMA PET/CT over conventional imaging for staging high-risk disease. Therapeutic applications followed, with the *VISION* trial (2022) establishing [^177^Lu]Lu-PSMA-617 as an effective treatment in patients with metastatic castration-resistant prostate cancer (mCRPC) after ARPI and taxane therapy, leading to FDA approval. The PSMAfore trial (2025) expanded this indication to ARPI-pretreated, chemo-naive mCRPC, confirming earlier use of PSMA radioligand therapy [34]. Future directions include evaluation in hormone-sensitive and neoadjuvant settings, exploration of alpha-emitting PSMA agents (^225^Ac, ^212^Pb), and integration of dosimetry and artificial intelligence for precision Theranostics [56,57,58,59,60].

A variety of α-emitting therapeutic agents have been investigated and are under evaluation for the treatment of metastatic prostate cancer, especially in the context of metastatic castration-resistant prostate cancer (mCRPC). Ongoing research continues to yield encouraging preliminary findings, suggesting potential efficacy in this challenging patient population Table 7.

Radium-223 dichloride (Xofigo^®^) is an alpha-emitting radiopharmaceutical characterized by a half-life of 11.4 days. Currently, it holds the distinction of being the sole agent approved by both the FDA and EMA for therapeutic use, although it is not classified as a theranostic agent. Functioning as a calcium mimetic, Radium-223 selectively targets bone metastases exhibiting heightened osteoblastic activity. Its clinical indication encompasses patients diagnosed with symptomatic, bone-predominant metastatic castration-resistant prostate cancer (mCRPC) in the absence of visceral metastases.

### 4.1. Global Disparities in Clinical Practice Patterns

Finally, there is a wide variation in practice patterns worldwide. A recent international survey revealed striking differences among centers in how patients are imaged, selected, and treated with [^177^Lu]Lu-PSMA therapy [45]. Such heterogeneity can lead to inconsistent outcomes and complicates the interpretation of trial data. Moving forward, greater standardization—through consensus protocols, harmonized imaging criteria, and structured training programs—will be vital to ensuring equitable access and reliable results across institutions [66].

### 4.2. Key Factors for Maintaining Variables Influencing SUV Measurements to Ensure Result Reproducibility

PSMA-PET/CT generates semi-quantitative biomarkers through standardized uptake values (SUV), including metrics like SUVmax, SUVpeak, PSMA tumor volume (PSMA-TV), and total lesion PSMA (TL-PSMA). These parameters serve not only for quantitative assessment but also hold potential as prognostic indicators. Consequently, it is imperative that these measurements exhibit both reliability and reproducibility for effective clinical application [67].

When evaluating lean body mass corrected standardized uptake values (SUV) versus body weight, various regions of interest (ROI) definitions have been utilized, including isocontour, fixed size, and maximum pixel methods. Although SUVmax is widely employed as a metric, using single pixel measurements can often be compromised in scenarios where the images exhibit significant noise. The statistical quality of imaging and the degree of noise are known to decline with reduced scan durations and lower activities of the evaluated lesions.

Factors influencing SUV (Standardized Uptake Value) measurements encompass several technical parameters. These include the radiotracer uptake time, which can affect the accumulation of the tracer in tissues, and baseline blood glucose levels in the context of FDG (Fluorodeoxyglucose) studies, as hyperglycemia can interfere with tracer uptake.

Body weight is another critical factor since SUV is normalized to patient weight. The injection technique employed can significantly impact local distribution and bioavailability of the radiotracer at the site of interest. Additionally, the calibration of the PET/CT camera is crucial for ensuring accurate quantification of uptake [68,69,70].

The partial volume effect must also be considered, as it can lead to underestimation of tracer activity in small lesions. Region of Interest (ROI) selection is pivotal, as improper delineation can skew results, and the reconstruction method used—including algorithms—can greatly affect image quality and quantitative outcomes. Finally, the matrix size used during the reconstruction process may influence the spatial resolution and thus the accuracy of SUV values.

To achieve optimal imaging outcomes, it is essential to standardize the protocols for both image acquisition and processing, while rigorously implementing quality assurance and quality control (QA/QC) measures.

To enhance the consistency of Standard Uptake Values (SUV) for both intra- and inter-study lesions, it is critical to employ a uniform scanner model that is properly calibrated. Additionally, utilizing the same imaging software and adhering to identical or closely related technical parameters will further strengthen measurement reliability.

Manuscripts detailing the findings of an investigational study should clearly outline all pertinent factors that may impact standardized uptake values (SUVs) within the Methods section. This is crucial for evaluating the comparability of the study’s results with the current body of literature and ensuring adherence to standardized practices in the field.

Taken together, these challenges underscore the need for a more precise, coordinated, and patient-centered approach. By addressing BCR more effectively, embracing individualized dosing, and aligning global practice standards, the field can move beyond its current limitations and continue to improve outcomes for patients with prostate cancer.

### 4.3. Expanded Approved Indication for [^177^Lu]Lu-PSMA-617 (Pluvicto^®^) in the Management of Metastatic Castration-Resistant Prostate Cancer (mCRPC)

Based on the results from the VISION trial involving a cohort of 831 men, there were significant improvements seen in both overall survival (median OS of 15.3 months versus 11.3 months, HR 0.62; *p* < 0.001) and radiographic progression-free survival. The trial also reported an acceptable safety profile and response rate. Consequently, the U.S. FDA first approved Pluvicto on 23 March 2022, for adult patients with PSMA-positive metastatic castration-resistant prostate cancer (mCRPC) who had previously been treated with both androgen receptor pathway inhibitors (ARPI) and taxane-based chemotherapy. Figure 4.

The eligibility criteria for the trial were strict, requiring evidence of disease progression after receiving at least one androgen receptor pathway inhibitor (such as enzalutamide or abiraterone) and one to two regimens of taxane chemotherapy. Confirmation of PSMA-positive lesions via PET imaging validated PSMA Theranostics as a standard of care in advanced prostate cancer, ultimately extending both survival and quality of life for men with limited treatment options.

Since the initial approval, the application of [^177^Lu]Lu-PSMA-617 radioligand therapy has been expanded based on the results from the Phase 3 PSMAfore trial [34], which enrolled 468 patients with PSMA-positive mCRPC who had progressed on ARPI and were suitable for deferring chemotherapy. On 28 March 2025, the FDA broadened the indication for Pluvicto to include adults with PSMA-positive mCRPC who had been treated with an ARPI and were considered appropriate candidates for delaying taxane-based chemotherapy. In this cohort, radiographic progression-free survival (rPFS) was 11.6 months for [^177^Lu]Lu-PSMA-617 compared to 5.6 months for an ARPI switch (HR 0.41; *p* < 0.0001). Although the overall survival (OS) in the interim OS analysis favored [^177^Lu]Lu-PSMA-617, the results did not reach statistical significance, partly due to crossover bias (HR 0.91). After adjusting for crossover, the HR was 0.59 [34]. These results served as basis for an expended FDA labeling issued on 28 March 2025. The 2025 expansion means that patients who have progressed on ARPI but haven’t yet received chemotherapy are now eligible for [^177^Lu]Lu-PSMA-617, enabling earlier therapeutic intervention with a favorable progression delay.

**Figure 8:**
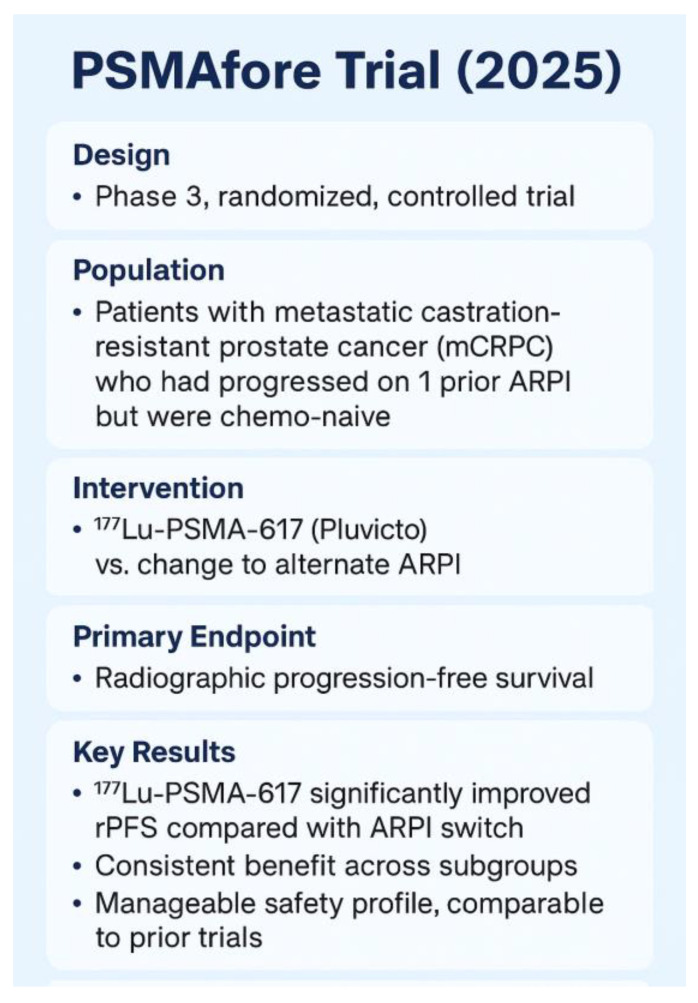
The Phase 3 PSMAfore trial (NCT04689828) [23,34].

The 2025 label expansion allows patients who have progressed on ARPI but have not yet received chemotherapy to be eligible for [^177^Lu]Lu-PSMA-617 (earlier use in chemo-naive mCRPC). This change facilitates earlier therapeutic intervention, resulting in a beneficial delay in disease progression Figure 8 [34].

### 4.4. Mechanisms of Resistance to PSMA-Targeted Radioligand Therapy

Resistance to PSMA-targeted RLT emerges inevitably, even in patients who initially respond well. Several overlapping biologicals, microenvironmental, and pharmacokinetic mechanisms have been characterized or hypothesized. A useful review is Stuparu et al. “Mechanisms of Resistance to Prostate-Specific Membrane Antigen–Targeted Radioligand Therapy” [71].

Key resistance pathways, their descriptions, supporting evidence, therapeutic implications, and combination strategies to overcome resistance are summarized in Table 8.

Mechanisms of Resistance to PSMA-Targeted Radioligand Therapy, key resistance pathways: A consequence of these is dose heterogeneity and suboptimal dosimetry—if the delivered absorbed dose to many tumor voxels is insufficient, residual disease may survive and regrow. Some trials are actively studying the correlation of lesion-level absorbed dose vs. response to refining this [72]. Combination Strategies to Overcome Resistance: Recognizing these resistance mechanisms, multiple rational combinations and sequencing strategies have been proposed or are in clinical development. These combination strategies aim to either sensitize tumor cells to radioligand therapy, broaden targetability, or suppress resistant clones Table 9.

PSMA loss and neuroendocrine differentiation represent significant mechanisms of treatment resistance that remain unresolved.

## 5. Future Directions

Prostate stem cell antigen (PSCA)-directed Chimeric antigen receptor (CAR) T cell therapies represent a promising area of investigation for men with metastatic castration-resistant prostate cancer (mCRPC). Currently, sipuleucel-T is the only immunotherapy demonstrated to extend survival in mCRPC. This autologous cellular vaccine activates dendritic cells to target prostate acid phosphatase [73]. However, there remains a significant need for more effective immunotherapies for mCRPC.

The only immunotherapy that has been shown to extend survival in metastatic castration-resistant prostate cancer (mCRPC) is sipuleucel-T, an autologous cellular vaccine that activates dendritic cells to target prostate acid phosphatase [73]. Despite this success, there is a pressing need for more effective immunotherapies for mCRPC.

The limited response to immunotherapy in prostate cancer is primarily due to significant tumor-induced immunosuppression. This immunosuppression restricts T cell trafficking and impairs effector function within the tumor microenvironment. Nevertheless, mCRPC expresses distinct tumor-associated antigens, such as prostate stem cell antigen (PSCA) and prostate-specific membrane antigen (PSMA), which present appealing targets for cellular therapies [74].

Inspired by the success of CAR T cell therapy in hematologic cancers, researchers have developed PSCA-targeted CAR T cells for prostate cancer. PSCA is highly expressed in advanced and bone-metastatic disease. Preclinical studies using second-generation PSCA-CAR T cells with 4-1BB co-stimulation have demonstrated potent and safe antitumor activity [74]. A first-in-human phase 1 trial evaluating PSCA-CAR T cells in mCRPC was reported in 2024. The primary endpoints of the study were safety and dose-limiting toxicities (DLTs) [75].

The authors reported no DLTs in the third dose level (DL3). Cytokine release syndrome of grade 1 or 2 occurred in 5 of the 14 treated patients. Furthermore, prostate-specific antigen levels declined by more than 30% in 4 of the 14 patients, along with radiographic improvements. The authors suggested that future clinical studies should optimize dosing and explore combination strategies to enhance durable therapeutic outcomes.

However, tumor antigen heterogeneity and treatment-emergent neuroendocrine (NE) transformation may represent broader mechanisms of resistance to CAR T therapy in advanced mCRPC [76]. As NE transformation becomes more common with the use of potent androgen receptor inhibitors, administering cellular immunotherapy earlier in the disease course may improve response durability. Additionally, dual targeting of antigens such as CEA or DLL3 could further mitigate antigen loss [77]. T cell exhaustion, indicated by PD-1 upregulation, and a high tumor burden may also limit efficacy [78]. Importantly, Checkpoint inhibition may restore CAR T cell activity in patients, leading to prolonged survival after subsequent pembrolizumab therapy.

Ongoing and upcoming clinical trials are expanding the role of PSMA-targeted theranostics beyond metastatic castration-resistant disease. Major studies such as UpFrontPSMA and PSMAddition are evaluating radioligand therapy in hormone-sensitive settings, while neoadjuvant applications are being actively explored. For example, Zhang and colleagues recently reported a proof-of-concept study investigating the use of ^177^Lu-PSMA as neoadjuvant therapy prior to radical prostatectomy in patients with high-risk metastatic hormone-sensitive prostate cancer. Their preliminary findings demonstrated encouraging responses, supporting the feasibility of incorporating radioligand therapy as a potential first-line neoadjuvant strategy [25]. Figure 7.

Beyond these efforts, earlier disease states such as oligometastatic and locally advanced prostate cancer are now being studied, raising the possibility that theranostics could shift from a palliative to a curative context [38,79,80,81] (Table S2). Parallel development of next-generation ligands and alpha-emitting agents (e.g., ^225^Ac, ^212^Pb-PSMA) [56,58,60] aims to increase therapeutic potency while overcoming resistance (Table 7). At the same time, advances in individualized dosimetry and artificial intelligence–driven quantification promise to optimize treatment delivery, improve safety, and further personalize therapy.

Together, these initiatives are poised to redefine the therapeutic landscape, moving PSMA theranostics earlier in the disease course and tailoring interventions to maximize benefit for individual patients.

## 6. Conclusions

PSMA-based imaging and radioligand therapy have revolutionized the management of prostate cancer. They provide more precise diagnosis and broaden treatment options for men with advanced disease. While [^177Lu^]Lu-PSMA-617 has demonstrated the clinical benefits of Theranostics, future advancements will rely on overcoming several key challenges. These include improving the management of biochemical recurrence, transitioning from fixed-dose regimens to individualized dosimetry, and standardizing practices across multiple centers internationally. Furthermore, the integration of advanced imaging technologies and artificial intelligence will enhance patient selection and monitoring. Collectively, these developments signal the onset of a new era in personalized care for prostate cancer, where Theranostics will increasingly play a critical role in optimizing patient outcomes.

## Figures and Tables

**Table 1:** Comparative Overview of Imaging Modalities in Prostate Cancer.

Imaging Modality	Mechanism/Tracer	Sensitivity (%)	Specificity (%)	Advantages	Limitations	Common Clinical Applications
Bone Scan (^99m^Tc-MDP)	Detects osteoblastic activity	65–80	60–75	Widely available; inexpensive	Low specificity; limited soft-tissue detection	Detection of bone metastases (advanced disease)
CT	Anatomical imaging; density-based	40–60	70–85	Rapid, widely used	Poor soft-tissue contrast; limited for early recurrence	Staging, structural assessment, response follow-up
MRI (with DWI)	Proton-based tissue contrast	75–85	80–90	Excellent pelvic imaging; multiparametric capability	Limited whole-body coverage	Local staging, detection of recurrence in prostate bed
Choline PET/CT	Radiolabeled choline metabolism	55–70	75–85	Detects biochemical recurrence	Reduced sensitivity at PSA < 1 ng/mL	Restaging after biochemical recurrence
PSMA PET/CT (e.g., ^68^Ga-PSMA-11, ^18^F-DCFPyL)	Targets PSMA expression	85–97	90–98	High lesion contrast; whole-body detection	PSMA-negative variants; access/cost	Primary staging, recurrence localization, therapy guidance
PSMA PET/MRI	Combines PSMA PET and MRI	90–98	90–98	High-resolution functional-anatomical data	Expensive; limited availability	Comprehensive staging, treatment planning

**Table 2:** Summary of Key PSMA PET Radiopharmaceuticals.

Tracer	Radionuclide	Main Advantages	Regulatory Status	Key References		
[^68^Ga]Ga-PSMA-11	β+ (68 min)	Rapid synthesis, high tumor-to-background ratio. Urinary clearance	FDA/EMA approved 2020	Eiber et al., 2015 [9]		
[^18^F]DCFPyL	β+ (110 min)	High yield, lower urinary excretion Higher resolution,	FDA approved—2021	Szabo et al., 2015 [10]		
[^18^F]rhPSMA-7.3 *	β+			Dual-labelling option (^18^F/^177^Lu); low renal uptake	FDA approved 2023	Jani AB et al., 2023 [11]
[^18^F]PSMA-1007	β+			Hepatic clearance; low bladder interference Less urinary activity, pelvic clarity	EU-approved Not FDA approved	Giesel et al., 2017 [12]
[^64^Cu]Cu-PSMA-617	β+ (12.7 h)	Delayed imaging, logistical flexibility	Investigational	Grubmüller B. et al., 2016 [13]		

* [^177^Lu]Lu-rhPSMA-7.3 has been replaced by [^177^Lu]Lu-rhPSMA-10.1.

**Table 3:** Common Toxicities of ^177^Lu-PSMA-617-Targeted Radioligand Therapy and Recommended Management Strategies.

Toxicity	Typical Incidence/ Severity (CTCAE Grade)	Mechanism & Risk Factors	Management Strategies
Xerostomia (dry mouth)	~30–60% Grade 1–2	Off-target uptake in salivary glands	Hydration; oral lubricants; sialogogues (pilocarpine, lemon drops); cooling packs during infusion; consider dose reduction for severe cases.
Fatigue	~30–50% Grade 1–2	Multifactorial (radiation effect, anemia, disease burden)	Energy conservation, rest scheduling, supportive care.
Nausea/vomiting	~20–40% (mild, transient) Grade 1–2	Short-term radiation-induced GI irritation	Antiemetics (ondansetron pre-/post-infusion); maintain hydration.
Hematologic toxicity (anemia, thrombocytopenia, leukopenia)	~10–20% Grade 2–≥3; higher with prior chemotherapy or marrow metastases	Marrow radiation exposure	CBC monitoring every 2–3 weeks; hold or reduce dose for grade ≥ 3; consider G-CSF support; transfusions as indicated.
Renal toxicity	5% (mild) Grade 1–2, rarely grade ≥ 3	Radiometal clearance via kidneys; cumulative dose	Monitor creatinine before each cycle; maintain hydration; dosimetry in high-risk; avoid nephrotoxic drugs.
Hepatic enzyme elevation	~10–15% (mostly grade 1–2)	Hepatic metastases or radiometal accumulation	Monitor LFTs; usually self-limited; hold therapy if >3× ULN.
Lacrimal gland irritation/epiphora	~5–10% Grade 1	PSMA uptake in lacrimal glands	Lubricating eye drops; cold compresses.
Pain flare (post-treatment)	<10%	Transient inflammatory response in bone lesions	Short-course corticosteroids or NSAIDs; self-resolves within days.
Long-term or rare effects	<1–2%	Possible cumulative marrow or renal dose	Periodic dosimetry and late toxicity follow-up.
Various—Severe toxicity (rare)	<5%	Grade 3–4	Usually after α-emitter therapy (^225^Ac-PSMA)

Abbreviations: G-CSF = granulocyte-colony stimulating factor; LFTs = liver function tests; ULN = upper limit of normal. Data sources: Derived from pivotal trials (VISION, TheraP) and institutional experiences. CTCAE: Common Terminology Criteria for Adverse Events: consistent with CTCAE v5.0 severity grading.

**Table 4:** Key Clinical Trials in PSMA Theranostics with safety data.

Trial (Year)	Agent	Phase	Population	Primary Endpoint	Key Efficacy Results	Safety Outcomes
VISION (2021) [23]	[^177^Lu]Lu-PSMA-617	III	mCRPC post-ARPI & chemo	OS, rPFS	OS + 4.0 mo; HR 0.62	53% Grade 3–4 AEs (anemia, thrombocytopenia, fatigue)
TheraP (2021) [27,33]	[^177^Lu]Lu-PSMA-617 vs. cabazitaxel	II	mCRPC	PSA50 response	66% vs. 37%	Less Grade 3–4 neutropenia vs. cabazitaxel
PSMAfore (2024) [34]	[^177^Lu]Lu-PSMA-617	III	mCRPC, ARPI-pretreated	rPFS	Median rPFS 12.0 vs. 5.6 mo	AE profile similar to VISION
SPLASH (2023) [35]	[^177^Lu]Lu-PSMA-I&T	III	mCRPC	rPFS	HR 0.71 vs. SOC	Mild-moderate hematologic AEs
ECLIPSE (2023) [36]	[^177^Lu]Lu-PSMA-I&T	III	mCRPC	PSA response	PSA50: 48%	Comparable safety to VISION
ACTINIUM (ongoing) [37]	[^225^Ac]Ac-PSMA-617	II	mCRPC (post-Lu)	PSA decline ≥ 50%	~65% PSA50	Xerostomia Grade 2 (65%), rare nephrotoxicity
SABR-PSMA (2023) [38]	[^177^Lu]Lu-PSMA-617 + SBRT	II	Oligometastatic PCa	PFS	Preliminary benefit	No unexpected toxicity
SAR-bisPSMA (2024) [39]	[^177^Lu]Lu-SAR-bisPSMA	I/II	mCRPC	Safety, efficacy	Ongoing	Favorable early safety
RPS-072 (2019) [40]	[^177^Lu]Lu-RPS-071	I/II	Advanced PCa	Safety, PSA50	Efficacy	Low-grade xerostomia, cytopenia

**Table 5:** Key Clinical Trials in PSMA Theranostics.

Trial	Year	Population	Intervention	Key Finding
proPSMA [8]	2020	High-risk PCa before curative Rx	PSMA PET vs. CT + bone scan	Higher accuracy, changed management
VISION [23]	2021	mCRPC, heavily pretreated	[^177^Lu]Lu-PSMA-617 + standard care	Improved OS and PFS
EMBARK [41]	2025	High-risk nmHSPC, rising PSA	Enzalutamide ± ADT	PSMA PET upstaged ~46% of patients

**Table 6:** Emerging Trends, AI Integration, and Future Directions in PSMA Theranostics.

Theme	Current Challenges	Emerging Solutions/AI Role	Key References
Quantification & Dosimetry	Variability in SUV/PSMA quantitation	AI-driven dosimetry and lesion segmentation	Hu J et al., [51]
Therapy Resistance	PSMA downregulation, neuroendocrine shift	Combination therapies (ARPI, PARP, immuno-, αβ-radiotherapy)	Rahbar et al., [47]
AI & Radiomics	Limited data standardization, overfitting risk	Multicenter AI models, federated learning	Sollini et al., 2019 [52]
Clinical Workflow Integration	Manual lesion annotation	Automated detection & reporting	Yazdani E et al., 2024 [53]
Ethics/Validation	Lack of regulatory framework	Transparent algorithms, reproducible datasets	IAEA AI-PET initiative, 2024- Artificial Intelligence [54]

AI limitations and solutions, combined future perspectives.

**Table 7:** Summary of key clinical studies using alpha-emitting PSMA-targeted radioligand therapy in metastatic castration-resistant prostate cancer (mCRPC).

Isotope/Agent	Study/Year	Design/Cohort	Median No. of Cycles (Dose)	PSA Response (≥50% Decline)	Median PFS/OS	Major Toxicities	Comments
^225^Ac-PSMA-617	Kratochwil et al., 2016 (JNM) [61]	First-in-human, single-center (*n* = 14)	3 cycles, ~100 kBq/kg every 8 weeks	70%	PFS ≈ 8 mo	Xerostomia (>80%), mild anemia	Proof-of-concept; high efficacy but dose-limiting salivary toxicity
^225^Ac-PSMA-617	Sathekge et al., 2019 (Eur J Nucl Med Mol Imaging) [62]	Prospective, post-Lu-PSMA cohort (*n* = 73)	Median 3 cycles, 100 kBq/kg	63%	PFS 9 mo; OS 15 mo	Xerostomia (78%), grade 3 anemia (12%)	Demonstrated efficacy in Lu-resistant disease
^225^Ac-PSMA-617 (TATCIST)	Ongoing, NCT05219500 [63]	Phase I/II multicenter dose-escalation	Up to 4 cycles	Pending	—	—	Evaluating optimal activity, dosimetry, and safety
^212^Pb-TCMC-PSMA	Stenberg et al., 2022 (Clin Cancer Res) [60]	First-in-human (*n* = 10)	2–4 cycles, 2.3–2.5 MBq/kg	60%	PFS ≈ 9 mo	Xerostomia (50%), mild cytopenias	Feasible with manageable toxicity
^212^Pb-J591	Tagawa et al., 2024 (JCO) [64]	Phase I, dose-escalation (*n* = 27)	Single or repeated cycles	41%	PFS ≈ 7 mo	Xerostomia, fatigue	Encouraging safety; planning phase II
^227^Th-PSMA-TTC	Morris et al., ClinicalTrials.gov [65]	Phase I, multicenter, dose-escalation (*n* = 65)	4 cycles, 10–20 kBq/kg	47%	PFS ≈ 8 mo	Fatigue (35%), anemia (20%), xerostomia (30%)	Promising antitumor activity; well-tolerated profile

List of α-emitting therapeutic agents that have been studied or are in clinical use/trials for the treatment of metastatic prostate cancer (primarily mCRPC—metastatic castration-resistant prostate cancer). Abbreviations: PFS = progression-free survival; OS = overall survival; TTC = targeted thorium conjugate; Lu = lutetium. Data sources: Selected peer-reviewed trials and ongoing multicenter studies through 2024.

**Table 8:** Mechanisms of Resistance to PSMA-Targeted Radioligand Therapy, key resistance pathways.

Mechanism	Description/Evidence	Implications for Therapy
Antigen loss or downregulation/heterogeneity	Some tumor clones lose or reduce PSMA expression (via transcriptional silencing, epigenetic changes, or clonal selection), making them invisible to the targeting ligand.	Leads to “cold” lesions not targeted by RLT. Suggests the need for dual antigen targeting, upregulation of PSMA (e.g., epigenetic agents), or combining with non-PSMA modalities.
Intratumoral heterogeneity & spatial/temporal discordance	Within a patient, lesions can differ in PSMA uptake, perfusion, or vascular delivery. Some metastases may be PSMA-negative or low expression (“mismatch lesions”).	Patients with discordant lesions are at higher risk of incomplete response; such heterogeneity limits uniform delivery of cytotoxic doses.
Radiation resistance/DNA repair upregulation	Surviving tumor cells may upregulate DNA damage repair pathways (e.g., nonhomologous end joining, homologous recombination) or adapt expression of repair proteins to survive sublethal radiation damage.	May require dose intensification, radiosensitizers, or combination with DNA damage response inhibitors (e.g., PARP, ATR, ATM inhibitors).
Hypoxia, poor vascular delivery, and microenvironmental protection	Hypoxic tumor regions are more radioresistant; poor perfusion limits radiopharmaceutical access; extracellular matrix, stromal cells, and immunosuppressive TME may shield tumor cells.	May benefit from modifying TME, using vascular normalization strategies, or combination with therapies that overcome hypoxia.
Efflux/pharmacokinetic barriers	Enhanced efflux of radionuclide-labeled ligand (via multidrug resistance transporters), increased ligand catabolism, or rapid clearance may reduce effective delivered dose.	Optimizing ligand chemistry, using alternate radionuclides or chelators, or combining with inhibitors of efflux (if safe) may help.
Clonal evolution & selection pressure	Therapy may select for resistant clones that pre-exist or evolve during treatment; these clones may have altered survival pathways, alternative antigen expression, or metabolic shifts.	Monitoring via sequencing, liquid biopsy, or repeat imaging can detect emerging clones and prompt early switch to alternate treatments.
Immune escape and tumor microenvironment suppression	The tumor may adopt immunosuppressive changes post-therapy, upregulate immune checkpoints (e.g., PD-L1) or recruit regulatory cells, which blunt potential bystander immune effects of radiation.	Suggests synergy with immunotherapy (checkpoint inhibitors, vaccines) to unmask immunogenic cell death.

**Table 9:** Combination Strategies to Overcome Resistance in PSMA-Targeted Radioligand Therapy.

Combination/Approach	Rationale & Preclinical/Clinical Evidence	Challenges & Considerations
PARP inhibitors/DDR inhibitors (e.g., PARP, ATR, ATM inhibitors)	Radioligand therapy induces DNA damage. Inhibiting repair pathways can potentiate lethal damage in tumor cells. Some preclinical and early clinical work supports synergy.	Risk of additive hematologic toxicity; optimal scheduling (concurrent vs. sequential) needs careful testing; patient selection (HRD mutation status) may modulate benefit.
Androgen receptor (AR) pathway inhibitors	AR inhibition may increase PSMA expression (PSMA is an AR-repressed gene) and sensitize to radiation. Some small trials and retrospective data have been explored by combining enzalutamide + ^177^Lu-PSMA.	Timing and sequencing are crucial; overlapping toxicities; risk of underdosing one modality.
Immune checkpoint inhibitors/immunotherapy	Radiation can induce immunogenic cell death and release neoantigens; combining with anti-PD-1/PD-L1 or anti-CTLA4 may enhance systemic anti-tumor immunity. The PORTER trial (Clinical and Translational Results) explores radiation + immune-activating agents.	Prostate cancer tends to be immunologically “cold”; risk of additive toxicity; selecting patients most likely to respond is key.
Dual radionuclide/tandem therapy (alpha + beta emitter combinations)	Combining a beta emitter (e.g., ^177^Lu) for broader coverage + alpha emitter (e.g., ^225^Ac) for high-LET cytotoxicity may overcome partial resistance and target differing tumor burdens. Anecdotal/early reports suggest similar PSA responses but improved PFS/OS.	Balancing toxicity (especially to salivary glands [59], bone marrow) is tricky; dose optimization and sequencing are still empirical; limited data so far.
Bispecific/multi-target agents/dual antigen targeting	Use of ligands or CARs or antibody–drug conjugates targeting PSMA plus alternate antigens mitigates antigen escape (loss of PSMA) by offering redundant targeting.	Complexity of engineering, safety, off-target toxicity; need of strong alternate antigen expression in tumor.
Epigenetic modulators/agents inducing PSMA re-expression	Drugs like HDAC inhibitors, DNMT inhibitors, or modulators of chromatin may upregulate PSMA expression in low-PSMA clones or reverse silencing. This can resensitize lesions to PSMA-RLT.	Timing and dose are critical; off-target effects on normal tissues; in vivo evidence is still emerging.
Vascular normalization/radiosensitizers/hypoxia-modifying agents	Agents like bevacizumab, anti–angiogenic, or hypoxia-targeting drugs (e.g., HIF inhibitors) can improve delivery or reduce hypoxia-induced radio resistance.	May alter tumor perfusion dynamics; risk of tissue injury; needs careful scheduling relative to radionuclide administration.
Sequential adaptive therapy/dose escalation based on dosimetry	Use of early imaging/dosimetry to identify underdosed lesions and adaptively boost or re-treat resistant foci.	Requires robust imaging infrastructure, dosimetry workflows, and regulatory flexibility; risk of cumulative toxicity.
AI-guided adoptive dosing	Personalized dosimetry and lesion-level response	Infrastructure, validation, cost

## Data Availability

No new data were created or analyzed in this study. Data sharing is not applicable to this article.

## Supplementary Materials

The following supporting information can be downloaded at: https://www.mdpi.com/article/10.3390/cancers18020234/s1, Table S1. Patient Selection, Dosing, and Monitoring Recommendations for PSMA-Targeted Radioligand Therapy; Table S2. Selected oligometastatic prostate cancer (OMPC) trials: metastasis-directed therapy and outcomes. Refs. [16,23,24,27,28,29,38,55,56,57,58,59,66,71,72,74,79,80,81] are cited in the Supplementary Materials.
