# Supplementary material for: PSMA-Based Radiopharmaceuticals in Prostate Cancer Theranostics: Imaging, Clinical Advances, and Future Directions
Source: Cancers (Basel). 2026 Jan 12;18(2):234. doi: 10.3390/cancers18020234 (PMC12838653; doi:10.3390/cancers18020234)
Supplement: Supplementary file 1 [file cancers-18-00234-s001.zip › cancers-3905023-supplementary.pdf]

Supplementary Materials

PSMA-Based Radiopharmaceuticals in Prostate Cancer Theranostics: Imaging, Clinical Advances, and Future Directions

Ali Cahid Civelek

1. Supplementary Essential Procedural Information for PSMA Radioligand Therapy (RLT)

1.1. Practical Guidance for PSMA-Targeted Radioligand Therapy (RLT)

1.1.1. Patient selection: (Table S1)

Table S1. Patient Selection, Dosing, and Monitoring Recommendations for PSMA-Targeted Radioligand Therapy.

Topic	Recommendation	Notes / Practical details
Indication	PSMA-positive mCRPC after progression on AR pathway inhibitors ± chemotherapy	Highest-level evidence population; PSMA PET ideally within 8–12 weeks of therapy.
PSMA PET selection	Lesions with uptake > liver; exclude dominant PSMA-negative / FDG-positive mismatch lesions	If discordant lesions exist, review in multidisciplinary board.
Performance status	ECOG 0–2 (0–1 preferred)	Frail patients require individual assessment.
Hematologic thresholds	Platelets $\geqslant 100\times10^9$/L; ANC $>1.5\times10^9$/L; Hemoglobin >9 g/dL	Lower values may be acceptable in selected cases with caution.
Renal function	Creatinine clearance ⩾50 mL/min (⩾ 30 mL/min with dosimetry)	Measured or Cockcroft–Gault estimate; adjust for extremes of body size.
Hepatic function	Bilirubin ⩽1.5×ULN; AST/ALT ⩽3× ULN (higher if extensive liver metastasis)	Interpret with context (e.g. Gilbert's).
Concomitant therapies & timing	Washouts for recent chemo or radium-223	Avoid additive marrow toxicity.
177Lu-PSMA-617 dosing	7.4 GBq every 6 weeks, up to 6 cycles	Adjust per toxicity; local practice allows 6–8.5 GBq.
225Ac-PSMA dosing	~100 kBq/kg per cycle every ~8 weeks; typically, 2–5 cycles	Use only at specialized centers; xerostomia is dose-limiting.
Monitoring	CBC & creatinine 2–3 weeks post-cycle; PSA pre-cycle	Imaging (PSMA PET or CT) after 2–3 cycles or per protocol.
Common toxicities & management	Myelosuppression, xerostomia, fatigue, nausea	Use dose delays/reductions, saliva substitutes, oral care.
Special considerations	Prior radium-223, heavy prior chemo, marrow involvement	Assess marrow reserve carefully; consider dosimetry oversight.

Abbreviations: ANC = absolute neutrophil count; ULN = upper limit of normal; mCRPC = metastatic castration-resistant prostate cancer [1].

Patients most suitable for PSMA-targeted radioligand therapy are those with PSMA-positive metastatic castration-resistant prostate

cancer (mCRPC) who have progressed after androgen receptor pathway inhibitors and/or chemotherapy, as demonstrated in the VISION trial [2]. Adequate PSMA PET expression is essential, typically defined as lesions with uptake above the liver and without discordant FDG-positive, PSMA-negative disease, which is associated with poor response (VISION, TheraP [3]. Eligible patients generally have ECOG performance status 0–2 and life expectancy greater than six months. Laboratory thresholds used in clinical trials include platelets $\geqslant 100 \times 10^9$/L, absolute neutrophil count >1.5 × 10^9/L, hemoglobin >9 g/dL, creatinine clearance $\geqslant$50 mL/min ($\geqslant$30 mL/min with caution), bilirubin $\leqslant$1.5 × ULN, and AST/ALT $\leqslant$3 × ULN. Prior exposure to chemotherapy, radium-223, or earlier RLT requires careful marrow reserve assessment. Patients with uncontrolled CNS disease or severe comorbidities are typically excluded. Standard counseling regarding contraception and radiation exposure applies [4].

1.1.2. Dosing and scheduling:

The approved regimen for ^{177}Lu-PSMA-617 is 7.4 GBq every six weeks for up to six cycles, as established in the VISION trial. Dose adjustment may be considered based on toxicity, body size, or institutional protocol (commonly 6–8.5 GBq). Individualized dosimetry can be employed to monitor absorbed doses to marrow, kidneys, and salivary glands. Laboratory monitoring should occur between cycles, with CBC and renal function checked at the expected nadir (approximately three weeks post-infusion). For alpha-emitting ^{225}Ac-PSMA therapy, dosing typically follows 100 kBq/kg every eight weeks for two to five cycles, primarily in patients progressing after ^{177}Lu therapy or requiring rapid cytoreduction [5] Xerostomia is the main dose-limiting toxicity, mitigated by dose reduction, increased dosing interval, or investigational protective strategies such as salivary cooling, botulinum toxin injection, or tandem ^{177}Lu + ^{225}Ac approaches. [6]

Prior therapies and timing: prior radium-223, recent myelosuppressive chemo, or prior RLT may require washout periods; careful marrow reserve assessment is essential. Several re-treatment trials require evidence of prior benefit and adequate recovery. [7]

Other considerations: symptomatic central nervous system disease, uncontrolled comorbidities, or pregnancy (for partners) are exclusions per usual nuclear medicine practice; counsel on contraception and radiation risk. [8]

1.1.3. 225Ac-PSMA (alpha emitter) when to consider and dosing caveats

- Where used: typically, in heavily pretreated mCRPC or after progression on 177Lu or when rapid cytotoxicity needed; not universally approved — mostly in specialized centers / trials/ compassionate use. [6]
- Common dosing schema from published series: ≈100 kBq/kg per cycle every ~8 weeks (many centers use this as a practical standard); typical total cycles 2 – 5 depending on response and toxicity.

Dose reduction or spacing used to manage xerostomia and marrow toxicity. Xerostomia is the dominant dose-limiting toxicity. [9]

- Strategies to reduce toxicity: consider tandem approaches (lower activity 225Ac + 177Lu), local salivary-gland protective measures (investigational: botulinum toxin injections, protective cooling — data mixed), and strict dose reductions for frail patients. These strategies remain under study [10]

1.1.4. Safety monitoring and supportive care

Before each treatment cycle, laboratory evaluation (CBC, creatinine, liver function tests) and symptom review should be performed. Clinicians should monitor for delayed myelosuppression (peak 2–6 weeks post-therapy), renal function decline, xerostomia, and fatigue. Grade ⩾3 toxicities should prompt treatment delay, dose reduction, or discontinuation. Supportive measures include saliva substitutes, meticulous oral hygiene, and referral to oral medicine specialists when appropriate. Ongoing multidisciplinary follow-up with oncology, nuclear medicine, and palliative care teams is recommended.

1.1.5. Global implementation

Successful program deployment requires regulatory readiness, stable isotope supply, trained staff, and defined referral pathways. National or regional authorization or inclusion within clinical trials is essential. Early payer engagement is recommended to address reimbursement challenges due to repeated dosing and upfront costs. Supply chain planning for ^{177}Lu and ^{225}Ac remains a critical barrier; hub-and-spoke radiopharmacy models and redundant supplier networks are advised. Facilities should ensure adequate shielding, validated radiopharmaceutical quality control, and appropriate waste management protocols. Workforce training and multidisciplinary tumor boards are central to safe implementation. Capacity planning should include estimation of treatment throughput, PSMA PET screening slots, and contingency for isotope shortages. [11]

1.1.6. Center start-up checklist

A new treatment site should adopt a standardized patient selection algorithm, secure reliable isotope supply, and establish written standard operating procedures covering radiopharmacy, quality control, radiation safety, and emergency handling. A multidisciplinary RLT tumor board (nuclear medicine, medical oncology, urology, radiation oncology, palliative care) should oversee patient management. Routine monitoring protocols for hematologic and biochemical parameters, PSA trends, and imaging should be implemented, alongside patient education on side effects and radiation precautions. Participation in registries or cooperative studies facilitates quality assurance and supports reimbursement negotiation. [12]

1.1.7. Future directions

Key priorities include individualized dosimetry to optimize tumor control while sparing organs at risk, rational sequencing and combination with AR-targeted agents, PARP inhibitors, chemotherapy, and immunotherapy, and evaluation of tandem or selective alpha-emitter strategies to minimize xerostomia. Broader global access will require validation of hub-and-spoke logistics, workforce training, and economic models adapted to diverse healthcare systems.

2. Oligometastatic Disease Management in Prostate Cancer

2.1. Oligometastatic Disease Management in Prostate Cancer

The concept of oligometastatic prostate cancer (OMPC) represents an intermediate disease state between localized and widespread metastatic disease, typically defined by the presence of ≤3–5 metastatic lesions detectable on advanced molecular imaging (e.g., PSMA PET/CT). This state is increasingly recognized as biologically distinct, with potentially indolent behavior and amenability to targeted, metastasis-directed interventions.

2.1.1. Diagnostic Refinement through PSMA PET Imaging

PSMA PET has revolutionized the detection of oligometastatic disease by uncovering lesions previously occult on conventional imaging. It allows for more accurate staging, patient stratification, and real-time assessment of disease burden, often reclassifying patients from "non-metastatic" to "oligometastatic" categories. This enhanced precision underpins the growing role of personalized, lesion-specific therapy.

2.1.2. Local and Metastasis-Directed Therapy (MDT)

Randomized trials such as STOMP and ORIOLE have demonstrated that stereotactic body radiotherapy (SBRT) to all PSMA-avid lesions can delay systemic therapy initiation and improve progression-free survival in OMPC (Table S2 Selected OMPC trials: metastasis-directed therapy and outcomes). In selected patients, MDT can achieve durable biochemical responses and defer androgen deprivation therapy (ADT), preserving quality of life. Combining MDT with systemic or radioligand therapies (e.g., Lu-177–PSMA-617) is an emerging strategy to eradicate micro-metastatic disease and consolidate local control.

2.1.3. Systemic and Theranostic Integration

In the setting of oligometastatic castration-resistant prostate cancer (omCRPC), theranostic approaches—particularly PSMA-targeted radioligand therapy (RLT)—have shown promise. Sequential or combined administration of SBRT and RLT may synergistically enhance efficacy by addressing both macroscopic and microscopic tumor sites. Prospective trials (e.g., PSMA-SRT, POPSTAR-2) are evaluating optimal sequencing, dosimetry, and response biomarkers.

2.1.4. Future Directions and Ongoing Trials

Emerging data suggests that molecular signatures (e.g., DNA repair gene alterations, AR-V7 status) and radiomics-based features from PSMA PET/MRI may refine patient selection for curative-intent MDT. Integration of theranostics, immunotherapy, and artificial intelligence–based response modeling will likely redefine the therapeutic landscape of OMPC in the next decade. Some of the current and ongoing clinical trials in oligometastatic prostate cancer are STOMP, ORIOLE, SABR-COMET [13], EXTEND, etc.

Table S2. Selected oligometastatic prostate cancer (OMPC) trials: metastasis-directed therapy and outcomes.

Trial / Name	Design / Population	Intervention (MDT or SBRT / MDT + systemic)	Primary Endpoint / Key Outcome	Results / Follow-up
STOMP (Surveillance or Metastasis-Directed Therapy for Oligometastatic Prostate cancer recurrence)	Phase II randomized; patients with biochemical recurrence after primary therapy; ⩽3 metastases on choline PET-CT; hormone sensitive. (BioMed Central)	MDT (SBRT or surgery) vs surveillance	ADT-free survival; also CRPC-free survival, overall survival (ASCOPubs)	At 5 years: ADT-free survival 34% in MDT vs 8% in surveillance; CRPC - free survival 76% vs 53%; OS ~85% in both arms at 5.3 years follow-up. (ASCOPubs)
ORIOLE (Observation vs SABR for Oligometastatic Prostate Cancer)	Phase II randomized; biochemically recurrent, hormone-sensitive prostate cancer; 1-3 metastases on conventional imaging. (JAMA Network)	SABR (complete consolidation of visible disease) vs observation	Progression at 6 months; PFS; local control; ADT-free survival; safety (PubMed)	Progression at 6 mos: 19% in SABR vs 61% in observation; median PFS not reached in SABR vs 5.8 months in control; no grade ⩾3 toxicities observed. (JAMA Network)
EXTEND	Randomized Phase II; oligometastatic prostate cancer (⩽5 metastases); comparison of radiation + intermittent hormone therapy vs hormone therapy alone. (MD Anderson Cancer Center)	MDT (radiation) plus intermittent ADT vs ADT alone	Progression-free survival; duration of hormone therapy break; tolerability (MD Anderson Cancer Center)	At median follow-up ~22.1 months, the median PFS had *not yet been reached* in the combined arm vs ~15.8 months in hormone-therapy only arm; the addition was well tolerated. (MD Anderson Cancer Center)

SABR-COMET: [13]

2.1.5. Commentary and Analysis:

- MDT delays systemic therapy: Both STOMP and ORIOLE show that metastasis-directed therapy in OMPC can substantially delay the initiation of androgen deprivation therapy. In STOMP, ADT-free survival increased from ~13 months (surveillance) to ~21 months with MDT (earlier reports) and maintained a favorable difference at 5 years. [14]

- Progression-free survival benefits: ORIOLE demonstrated a strong PFS benefit (not reached vs ~5.8 months) and a much lower progression rate at 6 months with SABR vs observation. [15]
- Safety profile: Across trials, MDT/SABR has had low rates of high-grade toxicity. In ORIOLE, no grade ⩾3 toxicities; in STOMP similar favorable toxicity. Thus, MDT in OMPC appears safe when used in well-selected patients. [15]
- Systemic therapy combinations: The EXTEND trial provides early evidence that combining radiation + intermittent hormone therapy improves PFS over hormone therapy alone and may allow longer hormone therapy breaks (which can be important for quality of life). [16]
- Imaging and biomarker enrichment: Several trials are integrating PSMA PET imaging, molecular/immune correlates (e.g. PSMA extracellular vesicles, T-cell receptor clonality) to better stratify patients likely to benefit. For example, ORIOLE's subset analyses show that complete consolidation of PSMA-PET-avid disease reduces new lesions at 6 months. [15]
- Gaps and future directions:
  - Larger phase III trials are needed to confirm survival benefit and long-term outcomes.
  - Optimal selection criteria (number of lesions, PSMA PET quantification, PSA doubling time) need standardization.
  - Studies of combining OMPC MDT with systemic or theranostic treatments such as PSMA-RLT are still sparse.
  - Cost-effectiveness, patient-reported outcomes, for instance quality of life, and effects on metastasis-free survival over long horizons are not yet fully defined.

3. Mechanism of resistance and combination strategies

3.1. Mechanisms of Resistance to PSMA-Targeted Radioligand Therapy

Resistance to PSMA-targeted RLT emerges inevitably, even in patients who initially respond well. Several overlapping biological, microenvironmental, and pharmacokinetic mechanisms have been characterized or hypothesized. [17].A useful review is Stuparu et al. "Mechanisms of Resistance to Prostate-Specific Membrane Antigen–Targeted Radioligand Therapy. [18].

Table 8 summarizes all major mechanisms of resistance to PSMA-targeted radioligand therapy, with concise descriptions and therapeutic implications.

A consequence of these is dose heterogeneity and suboptimal dosimetry — if the delivered absorbed dose to many tumor voxels is insufficient, residual disease may survive and regrow. Some trials are actively studying the correlation of lesion-level absorbed dose vs response to refining this [19]. UCSF Clinical Trials ID: NCT05435495]. UCSF Clinical Trials

3.2. Combination Strategies to Overcome Resistance

Recognizing these resistance mechanisms, multiple rational combinations and sequencing strategies have been proposed or are in clinical development. These combination strategies aim to either sensitize tumor cells to radioligand therapy, broaden targetability, or suppress resistant clones. Table 9 Summarizes the combination strategies to overcome resistance in PSMA-targeted radioligand therapy.

References

1. Uribe, C.; Iravani, A.; Savir-Baruch, B.; Jacene, H.; Graves, S.A.; Yuni, K.; Dewaraja, Courtney Lawhn Heath, and Thomas A. *Hope. J. Nucl. Med. Technol.* **2025**, *66*, 1528–1537.
2. Sartor, O.; de Bono, J.; Chi, K.N.; Fizazi, K.; Herrmann, K.; Rahbar, K.; Tagawa, S.T.; Nordquist, L.T.; Vaishampayan, N.; El-Haddad, G.; et al. Lutetium-177–PSMA-617 for Metastatic Castration-Resistant Prostate Cancer. *New Engl. J. Med.* **2021**, *385*, 1091–1103. https://doi.org/10.1056/NEJMoa2107322.
3. Karimzadeh, A.; Heck, M.; Tauber, R.; Solaris, E.; Nekolla, S.; Knorr, K.; Haller, B.; D'alessandria, C.; Weber, W.A.; Eiber, M.; et al. The Impact of PSMA PET–Based Eligibility Criteria Used in the Prospective Phase II TheraP Trial in Metastatic Castration-Resistant Prostate Cancer Patients Undergoing Prostate-Specific Membrane Antigen–Targeted Radioligand Therapy. *J. Nucl. Med.* **2023**, *64*, 1252–1258. https://doi.org/10.2967/jnumed.122.265346.
4. Hope, T.A.; Antonarakis, E.S.; Bodei, L.; Calais, J.; Iravani, A.; Jacene, H.; Koo, P.J.; Morgans, A.K.; Osborne, J.R.; Tagawa, S.T.; et al. SNMMI Consensus Statement on Patient Selection and Appropriate Use of ^{177}Lu-PSMA-617 Radionuclide Therapy. *J. Nucl. Med.* **2023**, *64*, 1417–1423. https://doi.org/10.2967/jnumed.123.265952.
5. Yadav, M.P.; Ballal, S.; Sahoo, R.K.; Tripathi, M.; Seth, A.; Bal, C. Efficacy and safety of ^{225}Ac-PSMA-617 targeted alpha therapy in metastatic castration-resistant Prostate Cancer patients. *Theranostics* **2020**, *10*, 9364–9377. https://doi.org/10.7150/thno.48107. PMID: 32802197; PMCID: PMC7415797.
6. Ma, J.; Li, L.; Liao, T.; Gong, W.; Zhang, C. Efficacy and Safety of ^{225}Ac-PSMA-617-Targeted Alpha Therapy in Metastatic Castration-Resistant Prostate Cancer: A Systematic Review and Meta-Analysis. *Front. Oncol.* **2022**, *12*, 796657. https://doi.org/10.3389/fonc.2022.796657.
7. National Cancer Institute. Re-Treatment with 177Lu-PSMA-617 for the Treatment of Metastatic Castration-Resistant Prostate Cancer, RE-LuPSMA Trial. 2025. Available online: https://www.clinicaltrials.gov/study/NCT06288113 (accessed on 12 December 2025).
8. Kratochwil, C.; Fendler, W.P.; Eiber, M.; Hofman, M.S.; Emmett, L.; Calais, J.; Osborne, J.R.; Iravani, A.; Koo, P.; Lindenberg, L.; et al. Joint EANM/SNMMI procedure guideline for the use of ^{177}Lu-labeled PSMA-targeted radioligand-therapy (^{177}Lu-PSMA-RLT). *Eur. J. Nucl. Med. Mol. Imaging* **2023**, *50*, 2830–2845. https://doi.org/10.1007/s00259-023-06255-8. PMID: 37246997; PMCID: PMC10317889.
9. Kratochwil, C.; Bruchertseifer, F.; Rathke, H.; Bronzel, M.; Apostolidis, C.; Weichert, W.; Haberkorn, U.; Giesel, F.L.; Morgenstern, A. Targeted α-Therapy of Metastatic Castration-Resistant Prostate Cancer with ^{225}Ac-PSMA-617: Dosimetry Estimate and Empiric Dose Finding. *J. Nucl. Med.* **2017**, *58*, 1624–1631. https://doi.org/10.2967/jnumed.117.191395.
10. Muniz, M.; Loprinzi, C.L.; Orme, J.J.; Koch, R.M.; Mahmoud, A.M.; Kase, A.M.; Riaz, I.B.; Andrews, J.R.; Thorpe, M.P.; Johnson, G.B.; et al. Salivary toxicity from PSMA-targeted radiopharmaceuticals: What we have learned and where we are going. *Cancer Treat. Rev.* **2024**, *127*, 102748. https://doi.org/10.1016/j.ctrv.2024.102748. PMID: 38703593; PMCID: PMC11160931.
11. Farolfi, A.; Armstrong, W.R.; Djaileb, L.; Gafita, A.; Hotta, M.; Allen-Auerbach, M.; Unterrainer, L.M.; Fendler, W.P.; Rettig, M.; Eiber, M.; et al. Differences and Common Ground in ^{177}Lu-PSMA Radioligand Therapy Practice Patterns: International Survey of 95 Theranostic Centers. *J. Nucl. Med.* **2024**, *65*, 438–445. https://doi.org/10.2967/jnumed.123.266391.
12. Herrmann, K.; Giovanella, L.; Santos, A.; Gear, J.; Kiratli, P.O.; Kurth, J.; Denis-Bacelar, A.M.; Hustinx, R.; Patt, M.; Wahl, R.L.; et al. Joint EANM, SNMMI and IAEA enabling guide: How to set up a theranostics

centre. *Eur. J. Nucl. Med. Mol. Imaging* **2022**, *49*, 2300–2309. https://doi.org/10.1007/s00259-022-05785-x. PMID: 35403861; PMCID: PMC9165261.

13. Palma, D.A.; Olson, R.; Harrow, S.; Gaede, S.; Louie, A.V.; Haasbeek, C.; Mulroy, L.; Lock, M.; Rodrigues, G.B.; Yaremko, B.P.; et al. Stereotactic Ablative Radiotherapy for the Comprehensive Treatment of Oligometastatic Cancers: Long-Term Results of the SABR-COMET Phase II Randomized Trial. *J. Clin. Oncol.* **2020**, *38*, 2830–2838. https://doi.org/10.1200/JCO.20.00818.
14. Ost, P.; Reynders, D.; Decaestecker, K.; Fonteyne, V.; Lumen, N.; De Bruycker, A.; Lambert, B.; Delrue, L.; Bultijnck, R.; Goetghebeur, E.; Villeirs, G.; et al. Surveillance or metastasis-directed therapy for oligometastatic prostate cancer recurrence (STOMP): Five-year results of a randomized phase II trial. *J. Clin. Oncol.* **2020**, *38*, 10. https://doi.org/10.1200/JCO.2020.38.6_suppl.10.
15. Phillips, R.; Shi, W.Y.; Deek, M.; Radwan, N.; Lim, S.J.; Antonarakis, E.S.; Rowe, S.P.; Ross, A.E.; Gorin, M.A.; Deville, C.; et al. Outcomes of Observation vs. Stereotactic Ablative Radiation for Oligometastatic Prostate Cancer: The ORIOLE Phase 2 Randomized Clinical Trial. *JAMA Oncol.* **2020**, *6*, 650–659. https://doi.org/10.1001/jamaoncol.2020.0147. PMID: 32215577; PMCID: PMC7225913.
16. Tang, C.; Sherry, A.D.; Haymaker, C.; Bathala, T.; Liu, S.; Fellman, B.; Cohen, L.; Aparicio, A.; Zurita, A.J.; Reuben, A.; et al. Addition of Metastasis-Directed Therapy to Intermittent Hormone Therapy for Oligometastatic Prostate Cancer: The EXTEND Phase 2 Randomized Clinical Trial. *JAMA Oncol.* **2023**, *9*, 825–834. https://doi.org/10.1001/jamaoncol.2023.0161.
17. Sengupta, S.; Kasireddy, S.R.; Mukkamala, R.; Srinivasarao, M.; Low, P.S. Prostate specific membrane antigen targeted PRMT5 inhibition re-sensitizes cancer cells to radiation therapies. *Cancer Res.* **2025**, *85*, 590. https://doi.org/10.1158/1538-7445.AM2025-590.
18. Stuparu, A.D.; Capri, J.R.; Meyer, C.A.; Le, T.M.; Evans-Axelsson, S.L.; Current, K.; Lennox, M.; Mona, C.E.; Fendler, W.P.; Calais, J.; et al. Mechanisms of Resistance to Prostate-Specific Membrane Antigen-Targeted Radioligand Therapy in a Mouse Model of Prostate Cancer. *J. Nucl. Med.* **2021**, *62*, 989–995. https://doi.org/10.2967/jnumed.120.256263. PMID: 33277393; PMCID: PMC8882874.
19. Thomas Hope. Mechanisms of Resistance to PSMA Radioligand Therapy-Study Prostate Cancer. **2025**. Available online: https://clinicaltrials.gov/study/NCT05435495 (accessed on 12 December 2025)
